# Trends and prospects in mitochondrial genome editing

**DOI:** 10.1038/s12276-023-00973-7

**Published:** 2023-05-01

**Authors:** Hong Thi Lam Phan, Hyunji Lee, Kyoungmi Kim

**Affiliations:** 1 Department of Physiology, Korea University College of Medicine, Seoul, 02841 Republic of Korea; 2 Laboratory Animal Resource and Research Center, Korea Research Institute of Bioscience and Biotechnology, 28116 Cheongju, Republic of Korea; 3 School of Medicine, Sungkyunkwan University, Suwon, 16419 Republic of Korea; 4 Department of Biomedical Sciences, Korea University College of Medicine, Seoul, 02841 Republic of Korea

**Keywords:** Genetic engineering, Experimental models of disease, Genetic techniques

## Abstract

Mitochondria are of fundamental importance in programmed cell death, cellular metabolism, and intracellular calcium concentration modulation, and inheritable mitochondrial disorders via mitochondrial DNA (mtDNA) mutation cause several diseases in various organs and systems. Nevertheless, mtDNA editing, which plays an essential role in the treatment of mitochondrial disorders, still faces several challenges. Recently, programmable editing tools for mtDNA base editing, such as cytosine base editors derived from DddA (DdCBEs), transcription activator-like effector (TALE)-linked deaminase (TALED), and zinc finger deaminase (ZFD), have emerged with considerable potential for correcting pathogenic mtDNA variants. In this review, we depict recent advances in the field, including structural biology and repair mechanisms, and discuss the prospects of using base editing tools on mtDNA to broaden insight into their medical applicability for treating mitochondrial diseases.

## Gene editing: moving into mitochondria

Applying gene editing techniques to the DNA found within mitochondria, the intracellular energy-producing organelles, is being explored as an approach to treating various diseases. Most of the cell’s genetic information is found in the nucleus, but mitochondria carry their own set of genes. Hong Thi Lam Phan Korea University, Seoul, South Korea, and coworkers review current trends and prospects in mitochondrial gene editing. Genetic defects in mitochondria have been implicated in many diseases, mainly affecting muscle and nervous tissues. A variety of methods for editing mitochondrial DNA are being explored, both as possible treatments and as methods to create animal models of human mitochondrial diseases. Challenges yet to be overcome, initially in animal studies, including finding effective means to deliver the molecular editing tools into mitochondria and achieving therapeutic results without harmful side effects.

## Introduction

**Fig. 1:**
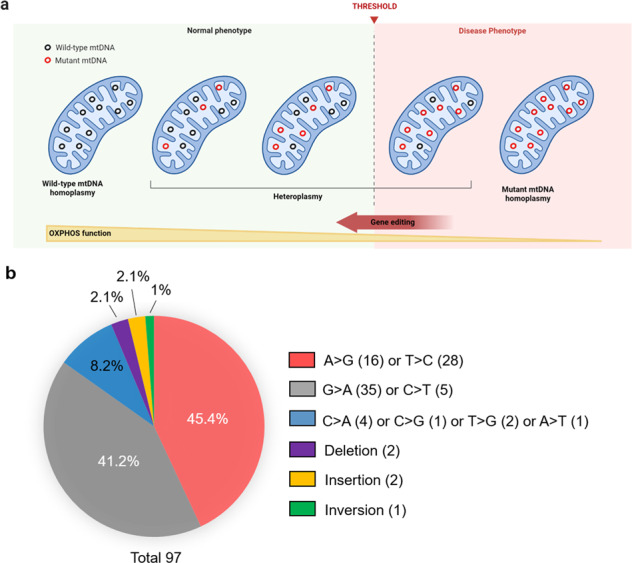
Distribution of the rate of human pathogenic mitochondrial DNA mutations. **a** The overall concept of heteroplasmic shifting. Although mutant and wild-type mtDNA can coexist, the exceeded threshold of mtDNA mutations compromises oxidative phosphorylation (OXPHOS). The mutations can be corrected by using gene editing platforms, which change the heteroplasmic level toward reducing the threshold. **b** Pie chart demonstrating the percentage of pathogenic mitochondrial DNA mutations in the MITOMAP database (accessed November 03, 2022). The white text reveals the distribution of transition point mutations, while the black text indicates transversion point mutations, deletions, insertions, or inversions. Parentheses indicate the number of pathogenic mitochondrial DNA mutations.

Mitochondria, double membrane-bound organelles referred to as the “powerhouses of the cell”, play an indispensable role in eukaryotic cells, as they are associated with metabolism^1,2^ and orchestrate a variety of other cellular functions, such as apoptosis, cell pluripotency, autophagy, calcium homeostasis, and innate immunity^3–9^. The mitochondrial genome is independent and distinct from that of the nucleus^10^. Human mtDNA, with a few hundred to several hundred thousand copies contained in each cell^11,12^, encodes 37 genes (22 tRNAs, 2 rRNAs, and 13 oxidative phosphorylation protein subunits) in a small circular 16.5 kb double-stranded piece of DNA^13,14^. The exceeded heteroplasmic threshold of mtDNA mutations manifests in multiple disorders (Fig. 1a)^14^. Specifically, a vast array of more than 250 mitochondrial defects have been implicated in various pathogenic mtDNA variants that primarily affect muscle and nervous tissues^15,16^. Notably, among the 97 known pathogenic mtDNA variants, point mutations accounted for 92 variants (approximately 95%), which indicated transition point mutations, including A > G, T > C, G > A, and C > T, accounting for 87% (MITOMAP: A Human Mitochondrial Genome Database. www.mitomap.org) (Fig. 1b). For instance, the *MT‑TL1* mutation m.3243 A > G can trigger multiple mitochondrial syndromes, such as chronic progressive external ophthalmoplegia (CPEO); maternally inherited diabetes and deafness (MIDD); and mitochondrial myopathy, encephalopathy, lactic acidosis, and stroke-like episodes (MELAS) syndrome. Meanwhile, three major mtDNA mutations, m.3460 G > A, m.11778 G > A, and m.14484 T > C, are present in more than 95% of patients suffering from Leber hereditary optic neuropathy (LHON), a maternally inherited disease that is associated with a loss of vision^17,18^. Myoclonus epilepsy and ragged-red fiber (MERRF) syndrome, a severe neurodegenerative defect, is predominantly caused by the point mutation m.8344 A > G in the *MT-TK* gene^19^. Furthermore, a spectrum of mutations in the *SPG7* gene, including c.861dupT, c.2221 G > A, c.2224 G > A, c.2228 T > C, c.1672A > T, c.1192 C > T, c.1529 C > T, c.1454_1462del, c.1067 C > T, c.184-3 C > T, c.233 T > A, c.1046insC, c.1457 G > A, and c.1053dup, were revealed in patients suffering from many mitochondrial diseases, such as progressive external ophthalmoplegia (PEO), ptosis, ataxia, spastic paraparesis, cerebellar atrophy, and proximal myopathy^20^. Strikingly, the C5024T mutation in the mtDNA gene *MT-TA* was uncovered via mouse models of heteroplasmic mitochondrial defects in the smooth muscle surrounding the colon and cardiomyocytes^21^. Although single-base substitutions can theoretically be corrected using base editors, precise and efficient therapeutics for several mtDNA mutations and mitochondria-involved diseases, have not been reported thus far. The development of and advances in base editing technology for correcting mtDNA base variants promise a potential therapeutic strategy for mitochondrial diseases and the generation of disease models in living cells and animals.

### The development of and advances in mitochondrial gene editing tools

Research on mitochondrial DNA editing started with the use of restriction endonucleases, such as PstI, SmaI, and ApaLI, engineered to be expressed exclusively in mitochondria (called mitoRes)^22–24^. The specific elimination of pathogenic mtDNA is followed by repopulation with normal mitochondrial DNA via heteroplasmy. Since mitoRes cannot be easily redesigned, it is difficult to use them to correct various clinical mutations, as there is the limitation of potentially editing the off-target loci^22–24^.

**Fig. 2:**
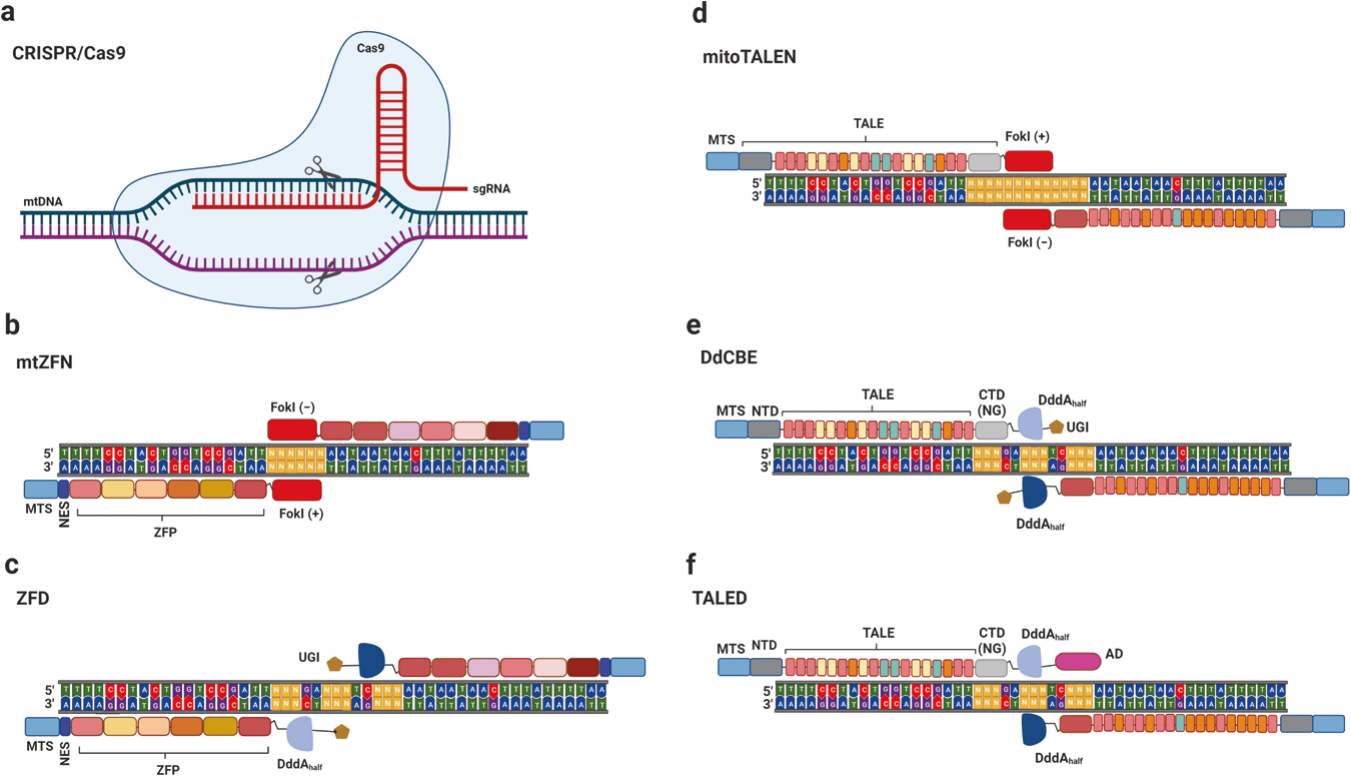
The architectures of several technologies for mitochondrial DNA editing. **a** A schematic of the architecture of CRISPR/Cas9 in the interactions with single-guide RNA and target DNA. The structures of **b** mtZFN, **c** ZFD, **d** mitochondrially targeted mitoTALEN, **e** DdCBE, and **f** TALED. ZFP zinc-finger protein, MTS mitochondrial targeting sequence, NES nuclear export signal, CTD (NG) C-terminal domain with NG repeat variable diresidues, NTD N-terminal domain.

Programmable DNA nucleases, such as clustered regularly interspaced short palindromic repeats (CRISPR)/CRISPR-associated protein 9 (Cas9), which recognize a short sequence known as the protospacer adjacent motif (PAM) and follow the DNA sequence targeted by a functional complex of Cas nucleases and a single guide RNA (sgRNA), has revolutionized gene modification and enabled the editing of mtDNA mutations^25–27^ (Fig. 2a). Of note, the CRISPR/Cas9 system, the most applied CRISPR construct, has recently been reported to efficiently cleave targeted mtDNA in HEK293T cells and zebrafish^26,27^. However, a key impediment to this approach is the import of the exogenous sgRNA into mitochondria^28^. Although several researchers have shown significant attempts at sgRNA delivery, this as-yet-unresolved limitation demonstrates the inefficiency of this approach^26,29,30^. Recently, one group demonstrated that not only Cas9 (type II) but also Cas12a (one of the type V CRISPR effectors) could access mitochondrial DNA editing^31^. However, although they tried to engineer the guide RNA using the RP loop related to importing mtRNA, clear evidence of efficient Cas12a-mediated mtDNA genome editing was not obtained^31^. Hence, to date, CRISPR-based systems have not been reliably applied to mtDNA manipulation.

Soon after the development of site-specific nucleases such as zinc-finger nuclease (ZFN) and transcription activator-like effector nuclease (TALEN)^32–34^, mitochondrially targeted ZFN (mtZFN) and mitochondrially targeted TALEN (mitoTALEN) versions emerged, optimized for delivery capacity into mitochondria, and they have been effectively used for heteroplasmic manipulation in several models of pathogenic mtDNA variants by inducing mtDNA double-strand breaks (DSBs)^35,36^. The sequence specificity of the mtZFN or mtTALEN monomers is demonstrated through interactions between the protein and DNA via tandemly organized repetitive elements derived from either zinc-finger protein (ZFP) or TALE DNA-binding protein (see Table 1 for a summary).

**Table 1:** Comparison of several mitochondrial DNA editing technologies.

Feature	mtZFN	mitoTALEN	CRISPR/Cas9	ZFDs	DdCBEs	TALEDs
Motif	ββα	RVD	Cas9/sgRNA	ββα	RVD	RVD
Binding	1 ZFP: 3 nucleotides (usually 3–6 ZFs)	1 TALE: 1 nucleotide (usually 13.5–18.5 RVDs)	1 sgRNA: 18–22 nucleotides	1 ZFP: 3 nucleotides (usually 4 ZFs)	1 TALE: 1 nucleotide (usually 15.5–18.5 RVDs)	1 TALE: 1 nucleotide (usually 10–20 RVDs)
Interaction	Protein–DNA	Protein–DNA	RNA–DNA	Protein–DNA	Protein–DNA	Protein–DNA
Recognition determinant	ZFP	TALE	sgRNA	ZFP	TALE	TALE
Edit element	FokI dimer	FokI dimer	Cas9	Split-DddA halves	Split-DddA halves	Split-DddA halves or inactive DddA_tox_, TadA
Correction of mtDNA deletions	Yes	Yes	Yes	No	No	No
Correction of mtDNA point mutations	Yes	Yes	Yes	Yes (C > T)	Yes (C > T)	Potentially (C > T and A > G)
AAV compatibility	One	Two	One	One	Two	Two
Off-target editing of mtDNA	High	High	Unknown	Low	Very low	Very low
Off-target editing of nuclear DNA	Not detected	Unknown	Unknown	Low	Relatively high but variable	Not detected
Applications	Human cells, mice	Human cells, mice	Human cells, mice, zebrafish	Human cells	Human cells, human embryos, zebrafish, mice, rats, and plants	Human cells
Advantages	Small	Relatively easy to design	Easy to design	Small, highly specific	Highly specific	Highly specific, easy delivery via AAV
Disadvantages	Reducing mtDNA copy number, relatively difficult to design, high cost, labor-intensive, time-consuming	Reducing mtDNA copy number, large, labor-intensive, time-consuming	Delivery	C-to-T conversion only	C-to-T conversion only, bystander editing, extensive off-target editing of nuclear DNA	Bystander editing

### Development of the structure and use of ZFP technology for mtDNA editing

Zinc-finger nucleic acid-binding modules are some of the most profuse proteins in eukaryotes and comprise the first programmable nucleic acid-binding domain applied in epigenome engineering^37–39^. Each ZFN strategy for nuclear DNA modification includes two DNA‐binding domains recognizing and binding a specific DNA sequence and a nonspecific FokI cleavage domain, which is a mediator for DNA excision and fragment insertion or frameshift variants of the DNA target (Fig. 2b). Structurally, the DNA-binding domain, consisting of three ZFP groups (each a 30-amino-acid module) bound to a 3-nucleotide DNA sequence, is composed of two adjacent β-sheets and an α-helix that interact with a single zinc atom, whereas the nuclease activity of the FokI cleavage domain, which must contain two distinct yet complementary parts, requires its dimerization^32^. Based on the favorable qualities of the components, the combination of the flexible and specific DNA-binding characteristics of ZFPs and the vigorous ablation ability of the FokI restriction enzyme in the ZFN architecture makes it credible for genome editing. To date, the potential of ZFN technology has been harnessed and effectively used for mtDNA manipulation. Initial reports revealed a site-specific methylase status using a three-finger monomeric ZFP equipped with an N-terminal mitochondrial targeting sequence (MTS) and a nuclear export signal (NES) fused to DNA methyltransferase 3a (DNMT3A), specifically targeting the m.8993 T > G mutation^40^. Furthermore, a single ZFP monomer conjugated to two FokI endonuclease domains tethered together via a flexible linker of 35 amino acids was engineered to target m.8993 T > G in heteroplasmic cybrid cells. However, this approach raises a safety concern that the combination of a four-finger ZFP monomer binding only 12 bp and a constitutively active nuclease might excessively reduce the mtDNA copy number^41^. However, attempts to improve its characteristics are still far from being efficient^42^.

Alternatively, optimizing the heterodimeric mtZFN architecture consisting of NARPd(+), an mtZFN specifically binding to a mutant sequence, and COMPa(−), a nonmutant sequence-bound mtZFN monomer, caused an effective shift in the heteroplasmy of cybrid cells bearing the m.8993 T > G mutation and “common deletions”^35^. Changes in the heteroplasmy shifting efficiency have also been demonstrated in a fine-tuned m.8993 T > G model^43,44^. Specifically, approximately 80% of the cybrid cells bearing the m8993T > G mutation were successfully transfected with the specific mtZFN that induced a considerable shift toward the wild-type mtDNA. Strikingly, substantial depletion of the mtDNA copy number was revealed at 24 h, followed by a return to a level comparable to nontransfected cells 28 days post-transfection. Moreover, the model was further capitalized to investigate the metabolic defects in mtDNA mutation-bearing cells^43^. In a later study^44^, the authors exploited a model called mTUNE to produce several isogenic cell lines (mT7, mT45, and mT80) with various levels of m8993T > G mutation (7%, 45%, and 80%, respectively). These m8993T > G heteroplasmic cell types augmented cell migration, a finding supported by reports of MDH1 increasing ATP generation via glycolysis.

Of interest, mtZFNs exert their potential applications in vivo^45^. In particular, a construct containing an m.5024 C > T-specific monomer (MTM25) and a wild-type-specific monomer (WTM1) was produced by encoding the monomers in a distinct adeno-associated virus (AAV) genome, followed by their encapsidation into AAV9.45 vectors subsequently administered to mice^21,45^.

Recently, a ZFP-based base editor for mtDNA called ZFD has been developed for catalyzing the conversion of C to T in human cells^46^. ZFD constructs were created by connecting ZFPs to the split interbacterial toxin deaminase DddA_tox_ and a uracil glycosylase inhibitor (UGI) via linkers (Fig. 2c). The optimized architecture of ZFDs showed successful base editing in HEK293T cells with frequencies of 2.6–30%^46^. Interestingly, the conversion of C to T mediated by ZFDs was performed in most Cs in both the TC and TCC sequence contexts. Although the rates of C to T conversion are currently low, further studies on engineering ZFDs to enhance both efficiency and precision may open a new door for correcting pathogenic mtDNA mutations in human embryos, fetuses, and specific adult tissues^46^.

### Development of the structure and use of TALEs technology for mtDNA manipulation

MitoTALENs have been platform-engineered via the fusion of the DNA-binding domain of TALEs, comprising monomers from the plant pathogenic bacteria genus *Xanthomonas*, and the nonspecific cleavage domain of FokI endonucleases^47,48^. The DNA-binding domain that recognizes the specific nucleotide in the target DNA sequence consists of tandem conserved repeats of 33-35 amino acids (Fig. 2d). Among them, two positions (residues 12 and 13), known as the repeat variable diresidue (RVD), show high variation in the recognition ability for a specific nucleotide^49^. Notably, 4 out of 25 RVDs (HD, NI, NG, and NN), which were the most prevalent, could identify C, A, T, and G, respectively^50^. In contrast to ZFNs, the structural foundation of the TALE DNA-binding specificity is more amenable to engineering and requires a minimal number of RVD panels encoding all base specificities^51^.

The first study on the development of mitoTALENs demonstrated the elimination of common deletions and the m.14459 G > A point mutation in mtDNA by applying two engineered constructs: Δ5-mitoTALEN and 14459A-mitoTALEN, respectively^36^; these architectures cause heteroplasmic shifting in heteroplasmic cybrid cells. Interestingly, a design based on the requirement of a T at position 0 in the sequence responsible for DNA recognition, called the T_0_ strategy, was used for m.13513 G > A mutation elimination in heteroplasmic cybrid cells^52^. However, attaining the binding specificity for a single nucleotide at a mutation position that is neither N > A nor N > T is currently far from easy. Furthermore, the T_0_ strategy requires two monomers, thus limiting the packaging capacity in various vectors, especially AAVs. To improve these hurdles, the authors showed that a shortened mitoTALEN monomer could also be applied for the efficient elimination of m.8344 A > G mutations. In addition, later studies depicted changes in the heteroplasmic shifting efficiency in iPSCs harboring m.13513 G > A and m.3243 A > G^53,54^. Treatment with mitoTALENs targeting the CD_5’_ region of mtDNA was shown to induce DSBs, resulting in a common deletion, followed by an accumulation of mtDNA^49,55,56^. In addition, human LHON m.14459 G > A and NARP m.9176 T > C mutations were specifically eliminated by injecting mitoTALENs into MII stage mouse oocytes^57^.

Interestingly, a monomeric variant of mitoTALENs known as mitoTev-TALEs, which are engineered by fusing a TALE-binding domain with a T4 phage-derived I-TevI homing endonuclease catalytic domain through a linker, has been used to manipulate m.8344 A > G mutant mtDNA in heteroplasmic cybrids^58^. However, this architecture has apparent disadvantages, such as requiring a CNNNG cleavage site for I-TevI.

To further explore the potential of TALE, hybrid platforms have been constructed by the fusion of solely mtDNA-binding TALEs and base-editing deaminases for precise modification of mtDNA. Recently, DdCBEs that can precisely and specifically convert C to T in human mtDNA have been formed by adding TALE proteins and UGI to split-DddA (Fig. 2e)^59^. Combining two TALE proteins with the mtDNA target resulted in the activation of the inactively nontoxic split-DddA halves. Surprisingly, the addition of UGI, protecting U from base excision by glycosylase, improved the efficiency of base editing by approximately 8-fold. Most excitingly, DdCBEs have been successfully exploited for mtDNA base editing in various species, such as human embryos, zebrafish, mice (embryos and adults), rats, and plants^60–68^.

Although the discovery of DdCBEs is a huge step forward, this technology is limited by the dependence of DddA on editable sites where Cs in the 5’-TC-3’ contexts are preferably converted to Ts (conversion from 5′-TCC-3′ to 5′-TCT-3′ or 5′-TTT-3′ is also available depending on the target). As a consequence, methods based on the original DdCBEs could correct only 10% of pathogenic mtDNA point mutations^69^. Soon after, the authors developed DddA variants using rapid phage-assisted continuous evolution. DddA6-containing DdCBEs conduct C•G to T•A conversion at TC sites 3.3-fold more efficiently than canonical DdCBEs. Furthermore, base editing with the DddA11 variant revealed strong compatibility over a range of settings (TC, AC, and CC), in which the editable levels for CC and AC in both the nucleus and mtDNA were increased by 15–30%^70^. Despite effective base-editing improvements, engineered DdCBEs are still limited to only being able to edit C-to-T residues. However, this limitation has been circumvented by the advent of TALEDs, a novel tool that enables the conversion of A-to-G^69^. This platform consists of an mtDNA-targeting TALE, adenosine deaminase TadA8e, and inactive DddA_tox_ as a cytosine deaminase (Fig. 2f). Moreover, TALEDs have been shown to successfully correct 47% of known pathogenic mtDNA point mutations in human cells^69^. More intriguingly, TadA8e, which supposedly operates on single-stranded DNA, was shown to possess an unexpected capability of adenine deamination in double-stranded DNA. This observation was explained by the presence of DddA_tox_, which enables the unwinding of the double-stranded DNA and provides TadA8e superfast access to the DNA for the necessary modifications^69^. However, this technology has the limitation of bystander editing, which converts nucleotides adjacent to the desired targets in the same editing window. Optimization in the engineering of DddA_tox_ or TadA might eliminate this undesired editing^69^. In addition, it would be intriguing to enhance the specificity and efficiency of TALEDs, which might provide further possibilities for mtDNA editing. Novel methods for the TALED-mediated manipulation of ES cells or direct editing in mouse embryos are certain to be developed in the near future.

### Off-target effects by mitochondrial gene editing tools

Solving unintended editing problems is of foremost importance for safe gene editing applications in clinical therapies. In the utilization of programmable editing platforms such as mtZFN and mitoTALEN, attenuating the temporary total mtDNA copy number is of paramount concern due to the depletion of mutant mtDNA before the repopulation of the wild-type mtDNA. Notably, the depletion might be significantly affected by the off-target editing of mtDNA as a consequence of inadequate reagent specificity or inappropriate concentrations of cleavage agents. Although off-target events of mtDNA editing using DdCBEs are relatively rare in HEK293T cells^59^, this platform can cause considerable mtDNA off-target editing in zebrafish and plants^62,65,66,68^. Most strikingly, two recent studies using DdCBEs have elucidated substantial off-target editing of the nuclear genome and mtDNA in mammalian cells and mice^71,72^. For instance, DdCBEs preferably caused undesired low-frequency mtDNA editing events at 5′-TC-3′ sequences, which were most prominent in the spacer regions to the left and right of the TALE binding sites, with some lying outside the regions^71^. Most unexpectedly, when delivered to fertilized 2-cell stage embryos by the genome-wide off-target analysis by two-cell embryo injection (GOTI) method, DdCBEs also induced remarkable sequence-independent off-target effects, resulting in single-nucleotide variants (1000–1500) in the nuclear DNA. The unexpected results are likely due to the unique characteristics of the DddA_tox_ cytosine deaminase used in DdCBE, which favors dsDNAs as substrates, unlike the substrate preference of cytosine deaminase APOBEC1 in the BE3 protein for ssDNA^71^. Further studies will be necessary to clarify the effects of various MTSs on off-target editing events by DdCBEs as well as to develop novel approaches to reduce the adverse effects. Using an unbiased method (Detect-seq) to evaluate genome-wide specificity, Lei. et al.^72^ demonstrated that hundreds of nuclear off-target edited sites were induced by DdCBEs in both TALE array sequence-dependent and sequence-independent cases. The studies suggested that certain interactions between the DdCBE and the CTCF binding region seem to be associated with sequence-independent off-target effects. However, the exact mechanism underlying this observation remains to be determined. Interestingly, this off-target editing issue was significantly improved by applying several advanced DdCBE constructs: UGI-NES-DdCBE, to which NES sequences were added to hinder the localization of DdCBE in the nuclei, and DddI_A_-DdCBE, in which DddI_A_, a natural immunity protein of DddA, was used to preclude nuclear DNA editing by DdCBE by obturating the active center of DddA_tox_^59,72^. Nonetheless, further thorough analyses of off-target editing of both mtDNA and nuclear DNA by various mtDNA editing technologies will be necessary for both basic research and clinical applications in the future.

## Conclusion

The potential of ZFPs and TALEs is promising, and their approaches for the base editing of mtDNA have been demonstrated. However, these platforms are limited by size compatibility, which makes their delivery to mitochondria less efficient using viral systems such as AAVs. In addition, there is a need for more efficient design and assembly methods for mtZFN and mitoTALEN monomers to recognize a wide spectrum of mtDNA sequences^52,73^. Consequently, the production of these proteins requires cost- and labor-intensive processes. These challenges could be overcome by using CRISPR/Cas9, a powerful editing system applicable to a wide number of organisms, including plants and mammals. CRISPR/Cas9 relies on only two components: (i) sgRNA, which recognizes a specific DNA sequence, and (ii) Cas9 nuclease, which cleaves the DNA sequence. However, as mentioned above, a major challenge in applying this system in mtDNA editing is to find effective methods for delivering exogenous sgRNA into the mitochondrion. If this could be achieved, the CRISPR/Cas9 system could potentially be broadly applied to manipulate mtDNA.

**Fig. 3:**
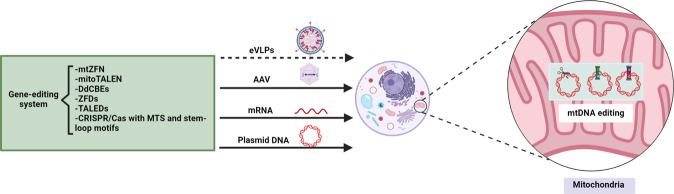
Strategies for delivering gene editing platforms into mitochondria. ZFP- and TALE-based gene editors or engineered CRISPR/Cas systems modified with MTS and stem‒loop motifs can be imported into mitochondria via transfection with AAVs or eVLPs, which encapsulate plasmid DNA or mRNA encoding the gene editing platforms. mtZFN, mitochondrial zinc-finger nuclease; TALEN transcription activator-like effector nuclease, DdCBE DddA-derived cytosine base editor, ZFD zinc-finger deaminase, TALE-linked deaminase (TALED); CRISPR/Cas clustered regularly interspaced short palindromic repeats/Cas.

In fact, several stem‒loop motifs mediating the shuttling of nuclear RNA molecules into mitochondria have been characterized at the 5′ or 3′ ends of a variety of endogenous RNAs, such as 5S rRNA, RNase MRP, and H1 RNA, which play an essential role in the functioning of mammalian mitochondria^74–76^. In particular, stem‒loop motifs from H1 RNA, which are appended to cytosolic RNA, facilitate the efficient importing of the RNA molecules into mitochondria^56^. Furthermore, it has been suggested that some 5S RNA motifs and two domains (yeast cytosolic tRNALys (CUU)-derived F-arm and D-hairpin) can also assist the uptake of synthetic RNA into mitochondria^77–79^. Moreover, it has been reported that the fusion of an MTS with the AAV2 capsid protein VP2 helped to target the NADH-encoding ND4 gene in mitochondria^80^. Strikingly, a recent study revealed that the use of an engineered Cas9 nuclease linked with N- or C-terminus MTSs for editing mtDNA mutations in HEK293T cells and zebrafish resulted in a significant reduction in mtDNA copy number^27^. These reports provide a critical proof of concept for the delivery of sgRNA appended to a stem‒loop element into mitochondria, followed by an interaction with the Cas9 nuclease and cleavage of specific mtDNA sequences. Moreover, not only Cas9 but also Cas12a can enter the mitochondria by using MTSs, and there have been attempts at mitochondrial delivery by engineering various stem‒loop motifs on guide RNAs^31^. Together, the studies suggest that efficient delivery of sgRNA via stem‒loop motifs show great promise for mtDNA-editing systems (Fig. 3).

Recently, a transient ribonucleoprotein (RNP) delivery platform using engineered virus-like particles (eVLPs) has been reported^81^ (Fig. 3). Essentially, eVLPs are empty viral shells into which therapeutic RNPs can be packed and delivered to the target DNA with minimal off-target editing effects^81^. Specifically, a newly engineered variant of eVLPs (v4 eVLPs) showed 16- and 4.7-fold increases in the packaging capacities of base editor RNPs and Cas9, respectively, compared to earlier architectures^82^. Most excitingly, eVLPs have been successfully used for delivering therapeutic packages to various organs in mice (liver, brain, and retina). These studies underscore the possibility of utilizing eVLP to deliver gene-editing cargoes for mtDNA to cells or multiple targeted organs in both animals and humans with minimal off-target editing^81^. Despite the potential of transient and efficient delivery of eVLPs, further studies on their pharmacokinetics, such as half-lives, are imperative. Additionally, determining the adequate requirements for base-editing cargoes may be a genetic therapy milestone for mtDNA editing in the future. Recently, extraordinarily novel applications based on both TALEs and ZFPs have been developed for highly specific mtDNA editing platforms toward heteroplasmic shifting with minimal cytotoxicity.

Optimizing the engineering of these platforms will surely pave the way for genetic approaches toward clinical trials by reducing the size to improve the packaging capability of viral systems and promoting efficiency and specificity for mtDNA manipulation. In fact, novel types of CRISPR systems, mini CRISPR-AsCas12f1 and miniature CRISPR-SpaCas12f1, consisting of 422 and 497 amino acids, respectively, can be feasibly and readily delivered to bacteria, cells, and tissues employing various delivery methods, such as AAV, plasmid, and RNP, have been reported^83,84^. Due to the advantages of editing efficiency and delivery, miniature platforms should also be considered an effective approach for mtDNA editing. Although the successful delivery of exogenous sgRNA into mitochondria using CRISPR‒Cas systems could prompt a revolution in mtDNA editing, it is undeniable that there are still real-world obstacles that need to be resolved for exceptional competence in the application of TALEs and ZFPs.
